# Relationship of cardiovascular disease risk factors and noncoding RNAs with hypertension: a case-control study

**DOI:** 10.1186/s12872-018-0795-3

**Published:** 2018-04-02

**Authors:** Shiying Chen, Rong Chen, Tingxing Zhang, Shaowei Lin, Zhou Chen, Bi Zhao, Huangyuan Li, Siying Wu

**Affiliations:** 1 Department of Epidemiology and Health Statistics, School of Public Health, Fujian Medical University, No. 1, Xueyuan Road, Fuzhou, 350122 Fujian China; 2 Department of Cardiology, The First Affiliated Hospital of Fujian Medical University, Fuzhou, 350005 Fujian China; 3 Department of Medical Management, Fujian University of Traditional Chinese Medicine Union People’s Hospital, Fuzhou, 350004 Fujian China; 4 Department of Nursing, Fuzhou General Hospital of Nanjing Military Command, Fuzhou, 350001 Fujian China; 5 Department of Preventive Medicine, Fujian Provincial Key Laboratory of Environment Factors and Cancer, School of Public Health, Fujian Medical University, No. 1, Xueyuan Road, Fuzhou, 350122 Fujian China

**Keywords:** Long noncoding RNAs, MicroRNAs, Cardiovascular disease risk factors, Essential hypertension

## Abstract

**Background:**

The present study sought to explore the relationship of common cardiovascular disease risk factors and noncoding RNAs with essential hypertension (EH).

**Methods:**

A total of 402 EH patients and 402 gender- and age-frequency matched healthy controls were enrolled in this study. Each participant received a questionnaire survey, physical examination and laboratory tests. Quantitative real-time polymerase chain reaction (qPCR) was performed to assess relative expression levels of six noncoding RNAs (NR_027032, NR_034083, NR_104181, miR-126, miR-143 and miR-145) in peripheral blood leucocytes. Multiple logistic regression analysis was used to estimate the risk of having EH between hypertensive and non-hypertensive patients.

**Results:**

Analysis showed that participants with anxiety, high body mass index, abdominal obesity and family history of hypertension had higher risk for EH, whereas those with bland diet and occupational physical activities had lower risk for EH. qPCR assays showed that NR_027032 (*P =* 0.015) and NR_034083 (*P =* 0.004) were significantly reduced in EH patients compared with controls, whereas NR_104181 (*P =* 0.007), miR-143 (*P =* 0.005) and miR-145 (*P =* 0.015) were significantly elevated.

After controlling the cardiovascular risk factors, multivariate analysis showed that lower expression levels of NR_034083 and higher expression levels of NR_104181 and miR-143 were risk factors for EH.

**Conclusions:**

EH is a result of environmental and epigenetic factors. Strikingly, NR_034083, NR_104181 and miR-143 may be correlated with the risk for EH development; therefore, epigenetic markers could be used to measure hypertension levels to help elucidate the pathogenesis of EH.

## Background

Hypertension, a leading risk factor for global cardiovascular disease, contributes to half of coronary heart disease and approximately two-thirds of cerebrovascular disease burdens [1]. While the pathogenesis of hypertension remains ambiguous, hypertension is generally believed to be a complex and multifactorial disease. Recent studies have revealed the relevance of noncoding RNAs with the pathogenesis of hypertension, supporting the importance of epigenomic regulation in hypertension progress [2, 3].

Currently, epigenetic regulators of hypertension mainly include long noncoding RNAs (lncRNAs), microRNAs (miRNAs) and DNA methylation [4]. LncRNAs have been recognised as critical regulators in the development of hypertension [5, 6]. Previous cell experiments have shown that NR_027032 regulates the function of vascular endothelial cells and is thought to be involved in vascular development, cell cycle, chromatin modification and DNA damage response genes [7]. NR_034083 is believed to regulate the proliferation, migration and phenotypic changes of vascular smooth muscle cells by regulating cell responses mediated by angiogenesis II [8]. NR_104181 has been shown to mediate rolling adhesion in Ca^2+^ and Ca^2+^ + Mg^2+^ and firm adhesion in Mg^2+^ and Mn^2+^, mimicking the two key steps in leucocyte accumulation in the inflamed vasculature [9].

MiRNAs are involved in a variety of biological processes, including cellular proliferation, apoptosis and differentiation [10, 11]. As the most highly enriched miRNA in endothelial cells, miR-126 can modulate human vascular endothelial phenotype in vitro and is required for the maintenance of vascular integrity and angiogenesis in vivo with a zebrafish model [12]. These findings were further confirmed by in vivo experiments with transgenic mice [10]. MiR-143 and miR-145 are co-transcribed in multipotent murine cardiac progenitors before becoming localised to smooth muscle cells and have been shown to cooperatively regulate the proliferation of vascular smooth muscle cells in transgenic mouse models [11, 13].

Despite the important role of noncoding RNAs (ncRNAs, including lncRNAs and miRNAs) in the development of hypertension, their relative contributions to hypertension remain largely unknown. Previous studies in humans have demonstrated that miRNAs (miR-126, miR-143 and miR-145) are differentially expressed in hypertensive patients and healthy individuals, though the expression levels of these miRNAs are not consistent among different reports [14–16]. There have been no previous studies on the expression levels of lncRNAs in hypertensive patient population samples. Thus, the goal of this study was to explore the relationship of cardiovascular disease risk factors and ncRNAs with essential hypertension (EH) in the population and propose countermeasures for this disease.

## Methods

### Study subjects

From November 2014 to June 2016, we performed a hospital-based case-control study on EH in Fujian Province, China. Participants were recruited from the First Affiliated Hospital of Fujian Medical University and the Affiliated People’s Hospital of Fujian University of Traditional Chinese Medicine. A total of 402 EH patients were included in the final analysis. Meanwhile, 402 healthy controls were randomly selected from the physical examination population in the same hospitals, and were frequency-matched by gender and age (±3 years) with cases. The disease-free status was ascertained according to the results of physical examination. Furthermore, 80 of the 402 EH patients and 80 of the 402 control individuals were selected by stratified random sampling method to donate venous blood samples for ncRNA expression analysis by quantitative real-time polymerase chain reaction (qPCR). Inclusion criteria for cases included patients with at least three consecutive records of systolic blood pressure ≥ 140 mmHg and/or diastolic blood pressure ≥ 90 mmHg or patients that have received antihypertensive medications for more than 3 months (European Society of Hypertension-European Society of Cardiology Guidelines, 2003). Inclusion criteria for controls included healthy individuals that have systolic blood pressure and diastolic blood pressure < 140 and < 90 mmHg, respectively. Exclusion criteria included a known history of leucopenia, thrombocytopenia or severe hepatic or renal dysfunction and evidence of an inflammatory or malignant disease. The protocol was approved by the ethics committee of Fujian Medical University School. Written informed consent was obtained from all patients and controls.

### Questionnaire survey

The participants completed the questionnaire themselves following instructions given by the researchers. The completed questionnaire was checked by the researchers to ensure that all questions have been answered appropriately. Questions covered demographic characteristics (e.g. gender, age, marital status, education level and occupation), lifestyle habits (e.g. tobacco smoking, alcohol drinking, diet, exercise, anxiety and depression levels) and family history of hypertension. Smokers were defined as individuals who smoked cigarettes more than once a day consecutively for more than 6 months or smoked more than 100 cigarettes cumulatively [17]. Alcohol drinkers were defined as subjects who had consumed any alcoholic beverage at least once per week for a minimum of 6 months [17]. A bland diet was defined as light food with no or very little oil, sugar, salt and spicy ingredients [17]. Occupational physical activities were classified into three categories depending on the intensity, duration and frequency of the participant’s physical activity at work. Light occupational physical activity was defined as 75% of the time for sitting or standing and 25% of the time for standing activities, such as office workers, salesmen and hotel waiters; moderate occupational physical activity was defined as 25% of the time for sitting or standing and 75% of the time for special activities, such as students’ daily activities, driving and electrical installation; and heavy occupational physical activity was defined as 40% of the time for sitting or standing and 60% of the time for special occupation activities, such as non-mechanised farming, dancing and mining [17]. The participants’ personality was classified into four types: participants with type A personality were defined as those who were always in a rush, highly productive, impulsive, stubborn and impatient; participants with type B personality were defined as those who were always in a soothing and self-regulating state; participants with type C personality were defined as those who always suppressed their emotions and had too much negative emotional experience; and participants with type D personality were defined as those who had the tendency to experience increased negative emotions most of the time and not share these emotions with others because of fear of rejection or disapproval [17]. Active exercise was defined as exercise lasting for at least 20 min once a week, whereas passive exercise was defined as exercise lasting less than 20 min once a week or no exercise at all [17]. Anxiety and depression were self-rated according to a previous report [17]. The self-rating anxiety scale (SAS) was defined as follows: < 50 normal, 50–59 mild anxiety, 60–69 moderate anxiety and ≥ 70 severe anxiety. The self-rating depression scale (SDS) was defined as follows: < 53 normal, 53–62 mild anxiety, 63–73 moderate anxiety and ≥ 74 severe anxiety.

### Physical examination

Systolic and diastolic blood pressure, height, weight and waist circumference (WC) were measured. Before blood pressure measurement, each subject was asked to not smoke, drink any beverage with caffeine or exercise for at least half an hour. After resting in a sitting position for 5 min, brachial blood pressure was measured three times with an interval of 2 min; the average of three readings was used for analysis [17]. Body mass index (BMI) was calculated as weight (kg) divided by the square of height in meters (m^2^) and categorised into four scales: < 18.5 underweight, 18.5–24.0 normal, 24.0–28.0 overweight and ≥ 28.0 obesity [17]. WC was classified into two categories: the waist of males < 85 cm or waist of females < 80 cm was considered normal, whereas waist of males ≥85 cm or waist of females ≥80 cm was considered abdominal obesity [17].

### Blood samples

Blood samples were collected in a K2-EDTA-coated tube (BD Biosciences, California, USA) and centrifuged at 400 *g* for 10 min to remove plasma. According to the manufacturer’s instructions for the Lymphocyte Separation Medium (Hao Yang Biological Manufacture Co., Ltd., Tianjin, China), blood samples were carefully transferred into a new RNA-free tube for a second centrifugation at 400 *g* for 20 min to obtain the peripheral blood leucocytes and then stored at − 80 °C prior to RNA extraction. All blood samples were subjected to only one freeze-thaw cycle.

### RNA extraction and qPCR

Total RNA was extracted from peripheral blood leucocytes using TRIzol reagent (Invitrogen, California, USA) and dissolved in RNase-free water. RNA extracts were quantified using NanoDrop 2000 instrument (Thermo Scientific, USA). The integrity of the RNA was determined by agarose gel electrophoresis. Reverse transcription of quantified RNA was performed using PrimeScript RT Reagent Kit with gDNA Eraser (lncRNAs) or the PrimeScript RT Reagent Kit (miRNAs) (Takara Bio Inc., Shiga, Japan) according to the manufacturer’s instructions. qPCR was performed on the LightCycler 480 real-time PCR system (Roche, Switzerland) with the SYBR® Premix Ex Taq™ II kit (Takara Bio Inc., Shiga, Japan). Amplification curves were obtained by 40 cycles of 95 °C for 30 s, 95 °C for 5 s and 60 °C for 34 s, whereas dissolution curves were obtained by one cycle of 95 °C for 15 s, 60 °C for 1 min and 95 °C for 15 s. The Ct value was the fractional cycle number at which the fluorescence exceeded the given threshold. GAPDH and U6 were used as internal controls. The reverse primer used for miRNA was a universal downstream primer. LncRNA and miRNA expression levels were calculated using the 2^-△△CT^ method [18]. The primers used for qPCR are listed in Table 1.

**Table 1:** List of the primers used for real time-PCR experiments

Noncoding RNAs	Oligo Name	Sequence (5′ to 3′)
GAPDH	Forward primer	GGACTCATGACCACAGTCCATGCC
	Reverse primer	TCAGGGATGACCTTGCCCACAG
NR_027032	Forward primer	GTCCTCCACTCCACCTCAAA
	Reverse primer	TGAGTTCCTGATCGTGTCCA
NR_034083	Forward primer	GGCTTCTAATCCGCCCTATC
	Reverse primer	CAATGACCCAAGGCAAATTC
NR_104181	Forward primer	CCATTCAGCTTTCACCATGTGC
	Reverse primer	ACCTTCAGGCGAGTCCAGATT
U6	Forward primer	GCTTCGGCAGCACATATACTAA
	Reverse primer	AACGCTTCACGAATTTGCGT
miR-126	RT primer	GTCGTATCCAGTGCAGGGTCCGAGGTATTCGCACTGGATACGACCGCATT
	Forward primer	GCGGCGGTCGTACCGTGAGTAA
miR-143	RT primer	GTCGTATCCAGTGCAGGGTCCGAGGTATTCGCACTGGATACGACGAGCTA
	Forward primer	GCGGCGGTGAGATGAAGCACTG
miR-145	RT primer	GTCGTATCCAGTGCAGGGTCCGAGGTATTCGCACTGGATACGACAGGGAT
	Forward primer	GCGGCGGGTCCAGTTTTCCCAG
Universal downstream primer	Reverse primer	ATCCAGTGCAGGGTCCGAGG

### Statistical analysis

All data were analysed using SPSS 22.0 (SPSS Inc., Chicago, IL, USA). Comparisons between the case and control groups were performed using a Z*-*test or Chi-square analysis. Multiple factor logistic regression was used to analyse whether lncRNAs and miRNAs were influencing factors of EH. All tests were two sided and a *P* value < 0.05 was considered statistically significant.

## Results

### Characteristics of the study population

Associations between demographic characteristics and EH are shown in Table 2. No significant difference was found in the general demographic characteristics between the case and control groups, including gender, age, marital status, education and occupation (all *P* > 0.05), indicating that the frequency matching was adequate. Associations between risk factors and EH are shown in Table 3. Compared with controls, EH patients had a lower bland diet rate (61.2% versus 75.6%, *χ*^*2*^ test, *P* <  0.001), higher abdominal obesity rate (84.0% versus 64.7%, *χ*^*2*^ test, *P* <  0.001) and higher family history of hypertension rate (47.0% versus 19.5%, *χ*^*2*^ test, *P* <  0.001). Significant differences were found between cases and controls in the occupational physical activities, personality types, anxiety levels and body mass indices.

**Table 2:** Demographic characteristics of hypertensive patients and control subjects

Characteristics		Hypertensive patients (*n* = 402)	Controls (*n* = 402)	*χ* ^*2*^	*P*
Gender	Male	187 (46.5)	187 (46.5)	0.000	1.000
	Female	215 (53.5)	215 (53.5)		
Age group (years)	< 45	22 (5.5)	22 (5.5)	0.000	1.000
	45–65	216 (53.7)	216 (53.7)		
	≥ 65	164 (40.8)	164 (40.8)		
Marital status	Single	4 (1.0)	2 (0.5)	0.850	0.660
	Married	363 (90.5)	369 (91.8)		
	Divorce/widowed	34 (8.5)	31 (7.7)		
Education level	Elementary degree and below	148 (36.8)	133 (33.2)	3.178	0.365
	Secondary degree	101 (25.1)	123 (30.7)		
	Senior/technical secondary degree	104 (25.9)	99 (24.7)		
	Associate degree and above	49 (12.2)	46 (11.5)		
Occupation	Worker	69 (17.3)	77 (19.2)	0.646	0.886
	Farmer	29 (7.3)	26 (6.5)		
	Retired personnel	181 (45.3)	177 (44.0)		
	Other occupation	121 (30.3)	122 (30.3)		

Data are presented as n (%)

**Table 3:** Comparison of risk factors between hypertensive patients and healthy controls

Risk factors		Hypertensive patients (*n* = 402)	Controls (*n* = 402)	*χ* ^*2*^	*P*
Smoking	Yes	87 (21.6)	102 (25.4)	1.556	0.212
	No	315 (78.4)	300 (74.6)		
Drinking	Yes	55 (13.7)	55 (13.7)	0.000	1.000
	No	347 (86.3)	347 (86.3)		
Bland diet	Yes	246 (61.2)	304 (75.6)	19.360	< 0.001
	No	156 (38.8)	98 (24.4)		
Occupational physical activities	Light	187 (46.5)	143 (35.6)	10.451	0.005
	Moderate	160 (39.8)	200 (49.8)		
	Heavy	55 (13.7)	59 (14.7)		
Exercise	< once/week	211 (52.5)	197 (49.0)	3.157	0.206
	1–2 times/week	14 (3.5)	24 (6.0)		
	≥ 3 times/week	177 (44.0)	181 (45.0)		
Personality type	A type	139 (34.6)	88 (21.9)	17.436	0.001
	B type	234 (58.2)	284 (70.6)		
	C type	22 (5.5)	26 (6.5)		
	D type	7 (1.7)	4 (1.0)		
Anxiety level	Normal	227 (56.5)	290 (72.3)	23.534	< 0.001
	Mild	140 (34.8)	95 (23.7)		
	Moderate	31 (7.7)	15 (3.7)		
	Severe	4 (1.0)	1 (0.2)		
Depression level	Normal	241 (60.0)	251 (62.4)	1.670	0.684
	Mild	119 (29.6)	104 (25.9)		
	Moderate	40 (10.0)	44 (10.9)		
	Severe	2 (0.5)	3 (0.7)		
Body mass index	< 18.50	8 (2.0)	23 (5.7)	46.103	< 0.001
	18.50–23.99	159 (39.6)	231 (57.5)		
	24.00–27.99	177 (44.0)	127 (31.6)		
	≥ 28.00	58 (14.4)	21 (5.2)		
Abdominal obesity	Yes	336 (84.0)	260 (64.7)	39.221	< 0.001
	No	64 (16.0)	142 (35.3)		
Family history of hypertension	Yes	189 (47.0)	78 (19.5)	68.349	< 0.001
	No	213 (53.0)	322 (80.5)		

Data are presented as n (%)

### LncRNAs and MiRNAs were differentially expressed in hypertensive patients and controls

When lncRNA levels between hypertensive patients and controls were compared, we found that NR_027032 (*Z-test* = − 2.439, *P* = 0.015) and NR_034083 (*Z-test* = − 2.890, *P* = 0.004) were significantly reduced, whereas NR_104181 (*Z-test* = − 2.685, *P* = 0.007) was significantly elevated in hypertensive patients compared with controls (Table 4). When miRNA levels between hypertensive patients and controls were compared, we found that miR-143 (*Z-test* = − 2.797, *P* = 0.005) and miR-145 (*Z-test* = − 2.436, *P* = 0.015) were up-regulated in hypertensive patients compared with controls, whereas miR-126 level (*Z-test* = − 1.679, *P* = 0.093) was not significantly different between hypertensive patients and controls (Table 4).

**Table 4:** Relative expression levels of lncRNAs and miRNAs in hypertensive patients and healthy controls (*P*_*50*_ (*P*_*25,*_ *P*_*75*_))

RNAs	Hypertensive patients (*n* = 80)	Controls (*n* = 80)	*Z*-test	*P*
NR_027032	0.839 (0.402, 1.485)	1.312 (0.493, 1.923)	−2.439	0.015
NR_034083	0.714 (0.513, 1.100)	0.940 (0.693, 1.588)	−2.890	0.004
NR_104181	1.946 (0.880, 3.013)	1.253 (0.568, 2.275)	−2.685	0.007
miR-126	1.751 (0.854, 2.904)	1.229 (0.548, 2.340)	−1.679	0.093
miR-143	1.796 (0.644, 3.470)	1.013 (0.420, 1.984)	−2.797	0.005
miR-145	1.708 (0.758, 2.945)	1.022 (0.585, 2.064)	−2.436	0.015

### NR_034083 was a protective factor for EH whereas NR_104181 and miR-143 were risk factors

We used EH as the dependent variable (0 = no, 1 = yes) and bland diet, occupation physical activities, personality type, anxiety level, BMI, abdominal obesity and family history of hypertension as independent variables for multivariate logistic regression analysis (assignment results are shown in Table 5, wherein personality type and BMI were used as dummy variables). After adjusting the demographic and environmental factors, logistic analysis showed that lower expression levels of NR_034083 (*OR* = 0.528, *95% CI* = 0.322–0.866) were a protective factor against hypertension, whereas higher expression levels of NR_104181 (*OR* = 1.651, *95% CI* = 1.164–2.342) and miR-143 (*OR* = 1.538, *95% CI* = 1.182–2.000) were risk factors (Table 6).

**Table 5:** Multivariate analysis variables and assignment

Variables	Assignment			
	0	1	2	3
Bland diet	No	Yes		
Occupational physical activities	Light	Moderate	Heavy	
Personality type	B type (0.0.0)	A type (1.0.0)	C type (0.1.0)	D type (0.0.1)
Anxiety level	Normal	Mild	Moderate	Severe
Body mass index	18.50–23.99 (0.0.0)	< 18.50 (1.0.0)	24.00–27.99 (0.1.0)	≥ 28.00 (0.0.1)
Abdominal obesity	No	Yes		
Family history of hypertension	No	Yes

**Table 6:** Multivariate logistic regression analysis of factors influencing the risk of hypertension

	*OR*	*95% CI of OR*	*P value*
Anxiety	2.737	1.344–5.574	0.006
Family history of hypertension	3.842	1.142–12.929	0.030
NR_104181	1.651	1.164–2.342	0.005
NR_034083	0.528	0.322–0.866	0.011
miR-143	1.538	1.182–2.000	0.001

## Discussion

In this case-control study, we first assessed the effects of common cardiovascular disease risk factors on hypertension in Fuzhou, China. Our study found that anxiety, BMI, abdominal obesity and family history of hypertension were risk factors for hypertension, whereas bland diet and occupational physical activities were protective factors for hypertension. Among the common risk factors, we found that higher levels of anxiety were associated with higher risk of hypertension. Anxiety may decrease the sensitivity of pressure reflex, leading to an increase of blood vessel tension and blood pressure as has been hypothesised previously [19].

In this study, we also assessed the association of six ncRNAs with hypertension and found that NR_027032 and NR_034083 were significantly reduced in hypertensive patients compared with control subjects. However, NR_104181, miR-143 and miR-145 were elevated in hypertensive patients compared with controls. After adjusting the demographic and environmental factors, multivariate analysis showed that lower expression levels of NR_034083 and higher expression levels of NR_104181 and miR-143 were risk factors for hypertension. In this study, the higher expression levels of miR-143 and miR-145 in hypertensive patients than in controls concur with previous reports from Paola Caruso et al. [14] and Santovito et al. [15] but are contradictory to the observations by Kontaraki et al. [16]. The absence of a significant difference in miR-126 expression levels between hypertensive patients and controls in our study is consistent with the report of Corsten et al. [20] but contrasts the report of Kontaraki et al. [21]. The reasons for these discrepancies are unclear but may be related to different blood samples (plasma [16], peripheral blood mononuclear cells [14, 21], peripheral blood leucocytes, peripheral total blood [20] and atherosclerotic plaques [15]), different disease processes (treated [15, 20] and untreated [16, 21] EH patients), different patient populations or undefined environmental factors. Clearly, further studies are needed to clarify these discrepancies.

Most importantly, we demonstrated for the first time that lncRNAs, similar to some miRNAs, are differentially expressed in hypertensive patients and healthy individuals, supporting their roles in the risk of human hypertension. The reduction of NR_034083 expression in hypertensive patients compared with controls suggests that this ncRNA is possibly a protective factor for hypertension. In support of this hypothesis, animal studies have demonstrated that NR_034083 is involved in negatively regulating angiotensin II [8], thereby leading to the development of hypertension. The up-regulation of NR_104181 expression in hypertensive patients suggests that this ncRNA is possibly a risk factor for hypertension, consistent with previous studies showing that NR_104181 can activate the function of lymphocytes, releasing a variety of inflammatory mediators to cause vascular dysfunction, including vasoconstriction, sodium and water retention and inhibiting the decrease of blood pressure [9, 22, 23].

Several limitations in our study need to be addressed. Firstly, recall bias was inevitable in this case-control study. However, it did not affect the ncRNA expression levels. Thus, it has a limited impact in studying epigenetic-disease association. Secondly, in the present study, blood samples collected can only present a single time point expression of lncRNAs or miRNAs. Thus, we did not look at the variability of the ncRNA expression level at different time points or under different conditions from the same individual [24]. Further mechanistic studies are needed to determine how these ncRNAs play their roles in hypertension. In addition, though the ncRNAs surveyed in this study, especially miR-126, miR-143 and miR-145, are known to be highly expressed in vascular endothelial and smooth muscle cells [14, 15], they might serve as attractive novel diagnostic biomarkers in peripheral blood leucocytes for human diseases.

## Conclusions

Our findings support previous observations of the effects of common cardiovascular disease risk factors on hypertension. In addition, our study demonstrated for the first time that lncRNAs (NR_027032, NR_034083, NR_104181), similar to some miRNAs, are differentially expressed in hypertensive patients and healthy individuals, supporting their roles in the pathogenesis of EH.

## Abbreviations

BMI: Body mass index
DBP: Diastolic blood pressure
EH: Essential hypertension
lncRNA: Long noncoding RNA
miRNA: MicroRNA
ncRNA: Noncoding RNA
SAS: Self-rating anxiety scale
SBP: Systolic blood pressure
SDS: Self-rating depression scale
WC: Waist circumference
